# Health-related quality of life and psychological distress among adults in Tanzania: a cross-sectional study

**DOI:** 10.1186/s13690-022-00899-y

**Published:** 2022-05-24

**Authors:** Olena Ivanova, Tembeka Sineke, Rebekka Wenzel, Elimina Siyame, Julieth Lalashowi, Abhishek Bakuli, Fidelina Cumbe Zekoll, Michael Hoelscher, Andrea Rachow, Denise Evans, Issa Sabi, Nyanda Elias Ntinginya

**Affiliations:** 1 Division of Infectious Diseases and Tropical Medicine, Medical Centre of the University of Munich (LMU), 80802 Munich, Germany; 2 German Center for Infection Research (DZIF), Partner Site Munich, 80802 Munich, Germany; 3 Health Economics and Epidemiology Research Office, Department of Internal Medicine, School of Clinical Medicine, Faculty of Health Sciences, University of the Witwatersrand, Johannesburg, South Africa; 4 Mbeya Medical Research Centre, National Institute for Medical Research (NIMR), P.O. Box 2410, Mbeya, Tanzania

**Keywords:** Quality of life, SF-36, K10, Mental health, Adults, Tanzania, Africa

## Abstract

**Background:**

Little data is available on health-related quality of life (HRQoL) and mental health of the general population in Tanzania. We aimed to describe HRQoL and level of psychological distress among adults in Mbeya and Songwe Regions of Tanzania.

**Methods:**

We conducted a cross-sectional study between April and October 2019 in Mbeya and Songwe Regions. Data were collected using the Medical Outcomes Short Form-36 (SF-36) questionnaire and the Page Kessler Psychological Distress Scale (K10). We described demographic characteristics of participants and used log-binomial regression to identify participant characteristics associated with psychological distress (K10 score ≥ 20).

**Results:**

A total of 393 adults were enrolled. The participants had a median age of 29 years (IQR 23–40) and 54.2% were male. Participants reported a physical component summary score (PCS) with a mean of 54.7 (SD7.1) and a mental component summary score (MCS) with a mean of 55.5 (SD5.1). Older participants (≥ 40 year) and those that were divorced/widowed reported lower physical functioning, energy/vitality and emotional well-being compared to their counterparts (*p* < 0.05). In terms of psychological distress, majority of participants (78.4%; 305/389) reported that they were likely to be well (K10 score < 20), while 13.4% (52/389) reported to have mild (K10 score 20–24), 5.7% (22/389) moderate (K10 score 25–29), and 2.6% (10/389) severe (K10 score ≥ 30) psychological distress.

**Conclusions:**

Physical function and mental well-being in this adult population from Tanzania were lower than that reported in other similar research in Tanzania and other African countries. This study provides valuable references for other research initiatives and clinical services in this region.

**Supplementary Information:**

The online version contains supplementary material available at 10.1186/s13690-022-00899-y.

## Background

The World Health Organization (WHO) defines health broadly as “*a state of complete physical, mental and social well-being and not merely the absence of disease or infirmity”*. [1] These different dimensions of health and well-being ensure that a population’s health is assessed in a subjective, reliable and more generalized way [2]. Health-related quality of life (HRQoL) is a comprehensive concept that includes domains related to physical, mental, emotional and social functioning [3]. This concept goes beyond direct measures of life expectancy and causes of death, and rather focuses on the impact, which health status has on individual’s quality of life. Population groups with different age, socio-economic or gender characteristics might define themselves as healthy or unhealthy in different ways depending on how they assess their health. Moreover, several factors, including environmental or socio-cultural factors, may affect an individual’s assessment of their health [4].

The use of self-assessed HRQoL measures has been recommended for monitoring health as a tool for disease risk screening and as standard part of clinical care [4]. Currently, a number of validated and standardized tools are available to assess HRQoL, among them is the Medical Outcomes Short Form-36 (SF-36) [5].

There have been efforts to estimate the quality of life of people in clinical settings and with different morbidities such as tuberculosis and HIV/AIDS in African countries. However, a vast majority of studies assessing HRQoL have a small sample size and rarely incorporate a control group from the general population [6–8]. Little is known about quality of life in general populations in these countries, including Tanzania. For instance, only one study from urban setting in Tanzania, conducted 30 years ago, assessed and validated SF-36 tool in general population [9]. The lack of HRQoL estimates and references for local “apparently healthy” populations might hinder the development of locally adapted health policies and strategies and make comparisons and the interpretation of research and clinical findings difficult.

One of the important components of HRQoL is the mental domain, and more specific instruments, such as the Page Kessler Psychological Distress Scale (K10), have been used to explore it [10]. However, a scarcity of evidence persists on the prevalence of mental disorders in the general population in low- and middle-income countries. Most studies to assess mental health in Tanzania are conducted in specific population groups such as HIV-positive people, elderly people, students, men who have sex with men, pregnant women and out- and inpatients [11–14].

This study was performed as a part of TB Sequel project, aimed at measuring the burden of lung impairment after tuberculosis (TB) and describing clinical, microbiological and socio-economic risk factors [15]. One of the specific objectives in the socio-economic sub-study of the TB Sequel was to describe HRQoL and mental distress in TB and post-TB patients in Africa. A number of systematic reviews suggested that the prevalence of depression among TB patients is relatively high [16] and TB has a substantial and encompassing impact on patients' quality of life [17]. Many TB survivors also face ongoing disability and increased mortality risks [18, 19]. However, in order to be able to quantify and to comprehensively examine the impact of active TB and post-TB impairment on HRQoL and mental health, there is a need for a proper comparison group from a general population with similar demographic and socio-economic characteristics.

## Methods

### Study aims

We aimed to describe health-related quality of life and level of psychological distress among adults in Tanzania. This data will serve as a reference for HRQoL and psychological distress and can be used to better understand and interpret results from clinical and epidemiological studies, including the TB Sequel Project [15], and also in clinical practice for patients with different morbidities in this area, including COVID-19 pandemic.

### Study design and sample

We used STROBE guidelines (checklist for cross-sectional studies) for this manuscript [20]. We conducted a cross-sectional study in Mbeya and Songwe Regions, in Southwestern Tanzania between April–October 2019. Mbeya Region is divided into five districts and has a population of 1,708,548, with the majority of population living in rural areas (1,021,973) versus urban (686,575) areas [21]. Songwe Region is divided into four districts and has a total population of 998,862, with mostly rural population (787,320) versus urban (211,53) population [22]. The two regions are considered to be moderately densely populated region compared to other regions in Tanzania. Literacy rate among the adult population is lower (82.9%, Mbeya regions and 74.6%, Songwe region) compared to other urban regions, e.g. 96% in Dar es Salaam [23]. The majority of the population have attended only primary school (89.8% in these areas) [21]. Unemployment rate in 2014 was 2,4%, comparable to the country average (2,6%) [23]. Leading causes of morbidity and mortality are malaria, pneumonia, clinical HIV/AIDS and upper respiratory tract infections [21].

Participants for the study were conveniently selected and they were mostly household members and neighbourhood contacts of the TB Sequel study participants [15], members of local communities and staff working at Mbeya Zonal Referral Hospital, where the TB Sequel study is taking place. This sampling method was chosen in order to provide comparison group for HRQoL and psychological distress for our TB and post-TB patients taking part in the TB Sequel study, which is conducted in Mbeya and Songwe Regions of Tanzania since 2017. The participants were directly recruited in the clinic, where TB Sequel patients were seen (relatives or family members) or in the communities, where TB Sequel patients lived. The sample size was selected to match the size of the TB Sequel cohort in Tanzania, around 400 recruited TB patients. Eligible participants were 18 years or older and were willing to provide informed consent for their participation in the study.

Potential participants were asked about their medical history. Adults with self-reported symptoms of TB (e.g., cough, weight loss, fever, night sweats, loss of appetite, fatigue, pain in chest), those with a history of TB, current or past respiratory diseases, cardio-vascular disease, previously diagnosed muscular disorder and other chronic conditions were excluded. Adults with a condition likely to lead to uncooperative behaviour (e.g., psychiatric morbidity or alcoholism) as well as current and active past smokers were also excluded. HIV and ART are known to have an impact on HRQoL [24]; however, because the HIV prevalence of HIV in Tanzania is low (4.7% among adults ages 15 to 49 years according to UNAIDS [25]) compared to countries in southern sub-Saharan Africa, such as Mozambique and South Africa, and many do not know their HIV status, it was not considered as an exclusion criterion.

### Data collection

Data were collected by experienced nurse through face-to-face interviews using SF-36 questionnaire for assessing HRQoL [5, 26, 27] and K10 for measuring psychological distress [10]. The SF-36 questionnaire has been validated for consistency and reliability in different settings for a multitude of health conditions [28–30]. Both questionnaires were applied mainly in English with oral translation to Swahili, where needed, by an experienced nurse from the TB Sequel Project [15]. Interview guides and training materials from TB Sequel were used as a support to ensure the consistency of data collection and its comparability [31].

All data were collected on paper forms and later captured in a clinical data management system - OpenClinica (community version) for data cleaning and analysis. Participant demographics, including sex, age, employment, education, marital status and HIV status were also collected.

### Measures

For the analysis, we scored the SF-36 according to the scoring guideline for this instrument [5, 26, 27]. For scoring, lower scores indicate more disability and higher scores indicate less disability i.e., a score of zero is equivalent to maximum disability and a score of 100 is equivalent to complete absence of disability [32]. The eight domains (i.e., physical functioning (PF), bodily pain (BP), role limitations due to physical health problems (RP), role limitations due to personal or emotional problems (RE), emotional well-being (MH), social functioning (SF), energy/fatigue (VT), and general health perceptions (GH)) were aggregated into two summary measures: the physical (PCS) and mental (MCS) component summary scores.

To measure mental health, we used the Page Kessler Psychological Distress Scale (K10) which is used as a screen to identify levels of psychological distress [10]. The questionnaire consists of 10 items about emotional states and each item is scored from one ‘none of the time’ to five ‘all of the time’. Scores of the 10 items are then summed, yielding a minimum score of 10 and a maximum score of 50. The scores are grouped into the following categories: likely to be well (scores < 20), likely to have a mild psychological distress (scores between 20–24), moderate psychological distress (scores between 25–29), and severe psychological distress (≥ 30).

### Statistical analysis

All analyses were carried out using STATA version 13 (STATA Corp, Texas, USA) and SAS version 9.3 (SAS Institute Inc., Cary, NC, USA).

First, to assess the quality of life and level of psychological distress among adults in Tanzania, we describe the eight domains of the SF-26, the two summary measures (PCS and MCS) and the proportion of adults reporting different levels of depression or anxiety. For the eight domains and the MCS and PCS summary scores, we present the mean, standard deviation, and Cronbach’alpha (> 0.70 was considered adequate) using the norm-based approach [32].

Second, to assess any differences in quality of life or level of psychological distress by demographic characteristics, we stratified the eight domains of the SF-36, the two summary measures (PCS and MCS) and the proportion of adults reporting different levels of psychological distress by participant demographics (i.e., age < 30, 30–34, 35–40, ≥ 40), sex (male and female), education (primary or lower, secondary, tertiary or higher), employment (employed, unemployed/student, other) and marital status (single, married/living with partner, divorced/widowed). To compare domains and summary scores across categories, we used the Kruskal–Wallis test for non-parametric data and the student t-test for parametric or normally distributed data.

Third, to identify participants who are more likely to report any form of depression or anxiety, we used a log-binomial regression model to test the association between participant characteristics and reporting any form of depression or anxiety (K10 score ≥ 20) [33]. We present the crude and adjusted relative risk (RR) and 95% confidence interval. Variables in the univariate model that were significant at the 0.2 level were included in the multivariate regression model.

## Results

A total of 393 adults were enrolled (Table 1). The participants had a mean age of 29 years (SD 12.7) and 54.2% were male. The prevalence of self-reported HIV was 2.5% (10/393).

**Table 1:** Characteristics of study participants and mental health (*n* = 393)

Characteristics	Females (*N* = 180)	Males (*N* = 213)	Total (*N* = 393)
**Age, years**			
< 30 years	87 (48.3)	118 (55.4)	205 (52.2)
30 to 34	17 (9.4)	35 (16.4)	52 (13.2)
35–39	19 (10.6)	14 (6.6)	33 (8.4)
≥ 40	57 (31.7)	46 (21.6)	103 (26.2)
**Marital Status**			
Divorced/widowed	29 (16.2)	7 (3.3)	36 (9.2)
Married/living with spouse	88 (49.2)	95 (44.8)	183 (46.8)
Single	62 (34.6)	110 (51.9)	172 (44.0)
**Level of education**			
Primary school or lower	91 (50.6)	88 (41.3)	179 (45.5)
Secondary education	46 (25.6)	70 (32.9)	116 (29.5)
University or higher	43 (23.9)	55 (25.8)	98 (24.9)
**Employment status**			
Employed	113 (62.8)	170 (79.8)	283 (72.0)
Unemployed or student	64 (35.5)	37 (17.4)	101 (25.7)
Other	3 (1.7)	6 (20.8)	9 (2.3)
**HIV Status**			
Negative	146 (81.1)	114 (53.5)	260 (66.2)
Positive	4 (2.2)	6 (2.8)	10 (2.5)
Unknown	30 (16.7)	93 (43.7)	123 (31.3)
**Psychological distress**			
Likely to be well K10 score < 20	131 (73.6)	174 (82.5)	305 (78.4)
Likely to have mild psychological distress K10 score 20–24	30 (16.9)	22 (10.4)	52 (13.4)
Likely to have moderate psychological distress K10 score 25–29	11 (6.2)	11 (5.2)	22 (5.7)
Likely to have severe psychological distress K10 score ≥ 30	6 (3.4)	4 (1.9)	10 (2.6)

### Quality of life and level of psychological distress

Participants reported a physical component summary score (PCS) with a mean of 54.7 (SD7.1) and a mental component summary score (MCS) with a mean of 55.5 (SD5.1). The SF-36 summary scores for eight domains were lower than those reported for adults randomly selected for the validation of SF-36 in urban Dar es Salaam, Tanzania (Table 2) [9].

**Table 2:** SF-36 scales aggregated for all participants (*n* = 393)

**SF-36 Norm-based scale**		**All (*n* = 393)**			Tanzania^*^ (*n* = 3802)	
	**Items**	**Mean**	**SD**	Alpha^**^	**Mean**	**SD**
Physical functioning (PF)	10	56.1	2.8	0.732	93.8	0.2
Role physical (RF)	4	54.6	8.3	0.682	84.2	0.5
Role emotional (RE)	3	52.9	10.6	0.681	88.2	0.3
Energy/vitality (VT)	4	61.8	6.2	0.695	74.9	0.3
Emotional well-being (MH)	5	55.1	6.9	0.715	82.2	0.3
Social functioning (SF)	2	53.6	6.6	0.648	88.2	0.3
Pain (BP)	2	59.1	7.7	0.661	82.8	0.4
General health (GH)	5	49.1	7.1	0.733	62.9	0.3
**Eight SF-36 scale scores**	36	219.3	17.8	n/a	DNA	DNA
Mental health component summary (MCS)	36	55.5	5.1	n/a	DNA	DNA
Physical health component summary (PCS)	36	54.7	7.1	n/a	DNA	DNA

*Legend*: *SD* Standard deviation, *DNA* Data not available, *n/a* Not applicable

*representative sample of an urban population in Tanzania [9]

**For the Alpha, Cronbach’ alpha value of > 0.70 was used to define good internal consistency of the SF-36 domains [24]

In terms of psychological distress, the majority of participants (78.4%; 305/389) reported that they were likely to be well (K10 score < 20) while 13.4% (52/389) reported to have a mild psychological distress, 5.7% (22/389) a moderate psychological distress, and 2.6% (10/389) severe psychological distress. Overall, one in five participants (21.6%; 84/389) reported any form of depression or anxiety (K10 score ≥ 20).

### Differences in quality of life or level of psychological distress by demographic characteristics

When stratifying by sex, the summary score of all eight SF-36 domains was lower among female participants compared to those among male participants (*p* = 0.165). When examining the individual domains, only physical functioning and pain were significantly lower among females (*p* < 0.001). When comparing the MCS and PCS summary scores, females had a lower PCS summary score compared to males (females PCS mean 54.7 SD 5.6; males PCS mean 56.2 SD 4.6; *p* = 0.044) (Table 3).

**Table 3:** Reliability, central tendency, and variability of scales among participants, stratified by sex (*n* = 293)

SF-36 Norm-based scale	Females (*n* = 180)			Males (*n* = 213)			*p* value**
	**Mean**	**SD**	Alpha*	**Mean**	**SD**	Alpha*	
Physical functioning	55.5	3.6	0.730	56.5	1.7	0.731	< 0.001
Role physical	53.9	9.4	0.699	55.1	7.2	0.652	0.158
Role emotional	52.8	10.9	0.679	53.0	10.2	0.671	0.978
Energy/vitality	61.5	6.3	0.693	61.9	6.2	0.688	0.498
Emotional well-being	54.5	6.9	0.725	55.5	6.9	0.694	0.126
Social functioning	53.1	6.9	0.654	54.1	6.3	0.629	0.241
Pain	58.0	8.5	0.664	60.1	6.8	0.651	< 0.001
General health	48.7	7.2	0.731	49.5	6.9	0.727	0.339
**Eight SF-36 scale scores**	216.6	19.1	n/a	221.5	16.3	n/a	0.165
Mental health component summary (MCS)	54.6	7.1	n/a	54.9	7.0	n/a	0.771
Physical health component summary (PCS)	54.7	5.6	n/a	56.2	4.6	n/a	0.044

^*^For the Alpha, Cronbach’ alpha value of > 0.70 was used to define good internal consistency of the SF-36 domains [24]

^**^For the p value, this was to indicate the difference between means for participants stratified by sex. *P* value < 0.05 was a cut-off for significance

Mean SF-36 scale scores varied with age category (Table 4). When stratifying by age, older participants (≥ 40 years) reported the lowest physical functioning (PF), energy/vitality (VT) and emotional well-being (MH) while younger participants (≤ 30 years) reported the highest (PF mean 54.9 SD 4.4 vs. 56.7 SD 1.4; VT mean 60.7 SD 7.2 vs. 62.7 SD 5.5; MH mean 53.7 SD 7.9 vs. 55.9 SD 6.4; *p* < 0.05). The combined SF-36 score was also lowest in older participants, and highest in younger participants (mean 217.2 SD 19.5 vs 220.7 SD 16.8; *p* = 0.07), respectively. We did not observe any differences in the MCS or PCS summary score across the different age categories (*p* > 0.05).

**Table 4:** Reliability, central tendency, and variability of scales among participants, stratified by age (*n* = 393)

SF-36 Norm-based scale	< 30 years (*n* = 205)			30 to 34 years (*n* = 52)			35 to 39 years (*n* = 33)			40 years and older (*n* = 103)			*p*-value**
	**Mean**	**SD**	Alpha*	**Mean**	**SD**	Alpha*	**Mean**	**SD**	Alpha*	**Mean**	**SD**	Alpha*	
Physical functioning	56.7	1.4	0.733	55.9	2.3	0.752	56.1	1.8	0.756	54.9	4.4	0.724	< 0.001
Role physical	54.5	8.8	0.712	55.5	5.8	0.719	55.1	7.1	0.676	54.1	8.8	0.639	0.8297
Role emotional	52.9	10.7	0.712	53.2	9.6	0.717	53.1	11.3	0.687	52.6	10.6	0.649	0.705
Energy/vitality	62.7	5.5	0.719	60.9	6.4	0.751	60.9	6.3	0.724	60.7	7.2	0.669	0.005
Emotional well-being	55.9	6.4	0.723	53.9	6.8	0.768	55.1	6.4	0.777	53.7	7.9	0.699	0.007
Social functioning	53.7	6.2	0.665	54	6.1	0.685	53.9	5.9	0.694	53.1	7.7	0.629	0.958
Pain	59.5	6.8	0.659	59.4	8.5	0.766	59.4	7.5	0.667	58.3	8.9	0.646	0.505
General health	49.7	6.9	0.746	47.7	7.6	0.783	47.8	6.8	0.754	49.2	7.4	0.718	0.187
**Eight SF-36 scale scores**	220.7	16.8	n/a	218.4	17.9	n/a	218.5	17.7	n/a	217.2	19.5	n/a	0.07
Mental health component summary (MCS)	55.2	6.9	6.9	54.4	6.9	n/a	54.8	6.5	n/a	53.9	7.6	n/a	0.383
Physical health component summary (PCS)	55.8	5	n/a	55.7	4.4	n/a	55.4	4.5	n/a	54.9	5.8	n/a	0.434

^*^For the Alpha, Cronbach’ alpha value of > 0.70 was used to define good internal consistency of the SF-36 domains [24]

^**^For the *p* value, this was to indicate the difference between means for participants stratified by age. *P* value < 0.05 was a cut-off for significance

Furthermore, when stratifying by marital status, divorced/widowed participants reported lower physical functioning (PF), energy/vitality (VT) and emotional well-being (MH) compared to married or single participants, respectively (PF mean 53.9 SD 4.9 vs. 56.1 SD 2.6 and 56.4 SD 2.1; *p* < 0.05) (VT mean 58.5 SD 7.9 vs. 61.9 SD 6.3 and 62.3 SD 5.6; *p* = 0.02) (MH mean 52.5 SD 6.9 vs. 55.3 SD 6.9 and 55.4 SD 6.9; *p* = 0.04) (Table 5).

**Table 5:** Reliability, central tendency, and variability of scales among participants, stratified by marital status (*n* = 391)

SF-36 Norm-based scale	Divorced/widowed (*n* = 36)			Married/living with partner (*n* = 183)			Single (*n* = 172)			*p* value**
	**Mean**	**SD**	Alpha*	**Mean**	**SD**	Alpha*	**Mean**	**SD**	Alpha*	
Physical functioning	53.9	4.9	0.643	56.1	2.6	0.753	56.4	2.1	0.725	< 0.001
Role physical	55.5	6.7	0.607	54.7	8.1	0.693	54.3	8.9	0.687	0.714
Role emotional	53.3	8.7	0.627	52.6	11.3	0.691	53.1	10.1	0.689	0.874
Energy/vitality	58.5	7.9	0.61	61.9	6.3	0.718	62.3	5.6	0.688	0.02
Emotional well-being	52.5	6.9	0.666	55.3	6.9	0.744	55.4	6.9	0.696	0.043
Social functioning	53.8	5.4	0.523	53.3	7.3	0.675	53.9	5.9	0.642	0.987
Pain	59.5	6.4	0.528	58.6	8.8	0.694	59.7	6.7	0.637	0.688
General health	49.2	6.5	0.669	49.4	7.5	0.742	49.5	7.9	0.748	0.773
**Eight SF-36 scale scores**	218.1	16.2	n/a	218.8	19.1	n/a	219.9	16.7	n/a	0.714
Mental health component summary (MCS)	53.3	6.8	n/a	54.7	7.3	n/a	55.1	7	n/a	0.179
Physical health component summary (PCS)	55.4	5.6	n/a	55.5	5.4	n/a	55.7	4.9	n/a	0.169

^*^For the Alpha, Cronbach’ alpha value of > 0.70 was used to define good internal consistency of the SF-36 domains [24]

^**^For the p value, this was to indicate the difference between means for participants stratified by marital status. *P* value < 0.05 was a cut-off for significance

When stratifying by the level of education (Supplementary Table 1), the only significant finding was that participants with primary or lower level of education (mean 48.7 SD 7.1) reported lower mean scores for general health compared to those with secondary level of education (mean 49.4 SD 7.9) or those with a tertiary or higher education (mean 50.8 SD 8.0); *p* = 0.007). Similarly, only the social functioning was significantly different when comparing the quality of life by employment status (Supplementary Table 2). Participants who were employed (mean 53.6 SD 6.7) and those who were unemployed/students reported (mean 54.4 SD 5.4) had higher mean scores compared to those who had another form of employment (mean 48.4 SD 9.9) (*p* = 0.016).

Using the Kessler psychological distress scale, the majority (78.4%) of all the participants were likely to be well with most scoring < 20 on the distress scale, however more females reported any form of psychological distress compared to males (score ≥ 20; 26.4% vs. 17.5%; *p* = 0.172). Among females, 16.9%, 6.2%, and 3.4% of participants reported mild, moderate, and severe forms of depression or anxiety, respectively while among males this was much lower (10.4%, 5.2% and 1.9% respectively) (Table 6).

**Table 6:** Psychological distress among participants (*n* = 389)

**Characteristics**		**All (*n* = 389)**				*p* value**
		**Likely to be well**	**Likely to have mild psychological distress**	**Likely to have moderate psychological distress**	**Likely to have severe psychological distress**	
		** < 20**	**20–24**	**25–29**	** ≥ 30**	
		**(*n* = 305)**	**(*n* = 52)**	**(*n* = 22)**	**(*n* = 10)**	
**Sex**						
Females	*n* = 178	131 (73.6%)	30 (16.9%)	11 (6.2%)	6 (3.4%)	0.172
Males	*n* = 211	174 (82.5%)	22 (10.4%)	11 (5.2%)	4 (1.9%)	
**Age**						
< 30 years	*n* = 204	170 (83.3%)	20 (9.8%)	10 (4.9%)	4 (2.0%)	0.075
30–34	*n* = 51	38 (74.5%)	9 (17.7%)	1 (1.9%)	3 (5.9%)	
35–39	*n* = 32	22 (68.8%)	5 (15.6%)	5 (15.6%)	0	
40 years and older	*n* = 102	75 (73.5%)	18 (17.7%)	6 (5.9%)	3 (2.9%)	
**Marital status**						
Single	*n* = 169	141 (83.4%)	19 (11.2%)	7 (4.1%)	2 (1.2%)	0.089
Married/living with partner	*n* = 182	139 (76.4%)	26 (14.3%)	10 (5.5%)	7 (3.8%)	
Divorced/widowed	*n* = 36	23 (63.9%)	7 (19.4%)	5 (13.9%)	1 (2.8%)	
**Education**						
Primary or lower	*n* = 178	125 (70.2%)	30 (16.9%)	15 (8.4%)	8 (4.5%)	0.014
Secondary	*n* = 116	100 (86.2%)	11 (9.5%)	3 (2.6%)	2 (1.7%)	
Tertiary or higher	*n* = 95	80 (84.2%)	11 (11.6%)	4 (4.2%)	0 (0%)	
**Employment**						
Employed	*n* = 279	218 (78.1%)	37 (13.3%)	16 (5.7%)	8 (2.9%)	0.910
Unemployed/student	*n* = 101	80 (79.2%)	14 (13.9%)	5 (4.9%)	2 (1.9%)	
Other	*n* = 9	7 (77.8%)	1 (11.1%)	1 (11.1%)	0 (0%)	

^**^For the *p* value, this was to indicate the difference between proportions using chi-squared statistic. *P* value < 0.05 was a cut-off for significance

Among participants that reported any form of psychological distress or anxiety (n = 84), 16.7% were younger than 30 years, 25.5% were between 30–34 years, 31.2% were between 35–39 years and 26.5% were 40 years and older. Although less than 10% of participants were divorced or widowed, reports of any form of depression or anxiety was highest in this group (13/36; 36.1%) compared to those who were married/living with a partner (43/182; 23.6%) or those who were single (28/169; 16.6%). Reports of any form of depression and anxiety were highest among participants who had a primary or lower education but was similar by employment. A similar proportion of participants with any form of depression or anxiety had a PCS score above (19.1%) or below (24.1%) the median cut-off (Table 6).

### Participant characteristics associated with any form of depression or anxiety

From the crude analysis, we found that females (RR 1.51 95% CI 1.03 – 2.21), those that were slightly older (35–39 years vs. < 30 years; RR 1.88 95% CI 1.03 – 3.41 and ≥ 40 years vs. < 30 years; RR 1.59 95% CI 1.02 – 2.48), those with a lower education level (primary or lower vs. tertiary or higher; RR 1.89 95% CI 1.12–3.16), and those that reported being divorced or widowed (divorced/widowed vs. single status; RR 2.18 95% CI 1.26 – 3.78) were more likely to report any form of depression or anxiety (K10 score ≥ 20) (Table 7). After adjusting for age, sex, marital status, and education, no association was found.

**Table 7:** Participant characteristics associated with any form of depression or anxiety

Characteristics		Proportion with outcome (*n* = 84); n/N (%)	Crude RR (95% CI)	*P* value (crude)	Adjusted RR (95% CI)	*P* value (adjusted)*
Sex	Female	47/178 (26.4%)	**1.51 (1.03–2.21)**	0.036	1.35 (0.90–2.02)	0.146
	Male	37/211 (17.5%)	1.0		1.0	
Age	< 30 years	34/204 (16.7%)	1.0		1.0	
	30–34	13/51 (25.5%)	1.53 (0.87–2.68)	0.138	1.53 (0.81–2.88)	0.190
	35–39	10/32 (31.3%)	**1.88 (1.03–3.41)**	0.039	1.70 (0.84–3.46)	0.140
	≥ 40 years	27/102 (26.5%)	**1.59 (1.02–2.48)**	0.042	1.23 (0.69–2.15)	0.475
Marital status	Single	28/169 (16.6%)	1.0		1.0	
	Married/living with partner	43/182 (23.6%)	1.42 (0.92–2.30)	0.104	0.92 (0.53–1.57)	0.753
	Divorced/widowed	13/36 (36.1%)	**2.18 (1.26–3.78)**	0.006	1.20 (0.57–2.53)	0.626
Education	Primary or lower	53/178 (29.8%)	**1.89 (1.12–3.16)**	0.016	1.76 (1.02–3.05)	0.432
	Secondary	16/116 (13.8%)	0.87 (0.93–2.19)	0.684	0.94 (0.48–1.85)	0.859
	Tertiary or higher	15/95 (15.8%)	1.0		1.0	
Employment	Employed	61/279 (21.9%)	1.0			
	Unemployed/student	21/101 (20.7%)	0.95 (0.61–1.47)	0.823		
	Other	2/9 (22.2%)	1.02 (0.29–3.52)	0.980		
PCS	Below cut-off (median)	47/195 (24.1%)	1.26 (0.86–1.85)	0.230		
	Above cut-off (median)	37/194 (19.1%)	1.0			

*Legend*: *RR* Relative risk, *CI* Confidence interval, *PCS* Physical component summary score

^*^The *p* values are obtained using a log-binomial regression model for the binary outcome of experiencing any form of depression or anxiety as yes or no. Bold *p* value < 0.05

## Discussion

This study described the quality of life and psychological distress among adults living in Mbeya and Songwe Regions, Tanzania. The results revealed low physical and mental component summary scores and showed that on average respondents scored lowest on general health and emotional issues followed by others. Furthermore, one in five (21.6%) participants reported any form of depression or anxiety disorder.

Scores in all eight domains (sub-scales) in SF-36 were lower comparing to the similar studies performed in Tanzania [9, 34]. After reviewing available literature on HRQoL in general population across African countries, we noticed that the scores in our population are also lower than those previously reported in Nigeria, Tunisia and Ghana [4, 35, 36]. The populations included in these studies are very heterogenous: urban citizens, people with non-communicable diseases or epilepsy, making direct comparisons to our findings for Mbeya and Songwe Regions difficult. Living standards and, therefore, quality of life is different between urban and rural African cities. Therefore, our findings provide useful estimates of quality of life and psychological distress for adults living in predominantly rural Tanzania. It is important to note that some participants selected for the current study were TB Sequel contacts. It is known that TB patients are more vulnerable, and that poverty (e.g., overcrowded living spaces, poor ventilation etc.) is a strong determinant of TB and its transmission [37]. Thus, it might be possible that by recruiting household members or neighborhood contacts of TB Sequel patients, our participants might come from settings with lower socio-economic status or may be more vulnerable to comorbidities and psychological stress. This may possibly explain why the HRQOL is lower than that reported for adults from Dar es Salaam [9]. These differences should be further explored by collecting detailed socio-economic, health and ethnicity data and performing qualitative research to obtain deeper insights into life circumstances and other relevant factors.

Our results support previous literature which has shown that in general females report poorer physical function compared to males [38–40]. Here we show that female participants had a lower PCS summary score compared to males. Older participants (≥ 40 years) also reported the lowest physical functioning (PF), energy/vitality (VT) and emotional well-being (MH) compared to younger age group. ﻿This effect of age was consistent with an expectation that health deteriorates with increasing age [41–43]. Conversely, being married potentially offers a protective mechanism against depressive symptoms and therefore against mental illnesses during late adulthood [44]. Furthermore, education has an influence on perceived quality of life. Participants with primary or lower level of education reported lower mean scores for general health compared to those with secondary or higher education. This trend was also observed in other studies from the general population [4, 45]. Thus, our results support the hypothesis that mental and physical scores are potentially higher in men, younger adults and people with higher education [9].

We used the K10 to measure psychological distress and showed that a significant proportion of participants (21.6%) in our study reported any form of depression or anxiety. This number correlates with other similar studies performed in students and HIV-positive people in Tanzania (15.5 – 21.9%) [12, 13], as well as in neighbouring country Uganda (17.4%) [46]. Although, there is limited research that explores correlates of poor mental health in African countries, including Tanzania, findings indicate that lower education level and female sex may be contributing factors [46–48]. Results from our crude analysis found that females, older age groups (≥ 35 years), divorced/widowed adults and participants with lower educational level were more likely to report any form of depression or anxiety.

### Limitations

It is worthwhile noting some of the limitations of the study. Firstly, the characteristics of our participants differ from the general Tanzanian population, i.e. self-reported HIV prevalence in our population is lower than previously reported for Tanzania (2.5% vs. 4.6%) [49] and Mbeya region (9%) [23]. Secondly, the health status of the participants was assessed subjectively using a questionnaire with a special focus on absence of respiratory or cardiac diseases. Thus, as many other studies using SF-36 questionnaire, we are relying on self-reported information on chronic or acute conditions or morbidities. Although, we have considered our participants to be “physically healthy”, there still might be a hidden undiagnosed burden of comorbidities influencing our findings. Thus, HRQoL scores and summary scores (MCS and PCS) reported in the study might be due to unrecognized clinical conditions interfering with everyday life of participants. In addition, as some of our participants are coming from households or the same environment as TB Sequel patients, they could be mentally affected by the burden of TB in their immediate environment. Thirdly, the K10 is a screening tool and is not a clinical diagnosis of mental health disorders such a depression or anxiety, therefore in the absence of a clinical diagnosis the K10 score may over or under-estimate mental illness in the sample. Lastly, the questionnaire was primarily designed in English and verbally translated to the participants by the trained staff, which could lead to some bias related to understanding of the questions. Moreover, half of the participants had primary or lower levels of education, which might also interfere with the understanding of some terms or questions and affect self-reported responses regarding potential comorbidities and clinical diagnosis.

## Conclusions

To our knowledge, this was the first study to measure HRQoL and psychological distress in a population, that reported no respiratory or cardio-vascular diseases in Southwestern Tanzania. Physical and mental health in adult population in this study was lower compared to other similar research in Tanzania and other African countries. ﻿Socio-demographic factors such as gender, age and education should be considered during the development and implementation of health policies and strategies aiming to improve health and well-being of the local population. Thus, it is important to undertake a larger assessment of quality of life and psychological distress in a representative sample (urban/rural; different socio-economic status; educational levels etc.) of the general Tanzania population (for example, during national surveys). Nevertheless, this study provides valuable references for other research initiatives and clinical services in this region.

## Supplementary Information

**Additional file 1**.

## Data Availability

The data supporting the findings are available upon reasonable written request to the corresponding author.

## Abbreviations

AIDS: Acquired immunodeficiency syndrome
BP: Bodily pain
HIV: Human Immunodeficiency Virus
HRQoL: Health-related quality of life
GH: General health perceptions
K10: Page Kessler Psychological Distress Scale
MCS: Mental component summary score
MH: Emotional well-being
PCS: Physical component summary score
PH: Physical functioning
RE: Role limitations due to personal or emotional problems
RP: Role limitations due to physical health problems
SF-36: Medical Outcomes Short Form-36
SF: Social functioning
VT: Energy/fatigue
