# Supplementary material for: Health-related quality of life and psychological distress among adults in Tanzania: a cross-sectional study
Source: Arch Public Health. 2022 May 24;80:144. doi: 10.1186/s13690-022-00899-y (PMC9127286; doi:10.1186/s13690-022-00899-y)
Supplement: Supplementary file 1 — Additional file 1. [file 13690_2022_899_MOESM1_ESM.docx]

**Supplement**

**Supplementary Table 1.** Reliability, central tendency, and variability of scales among participants, stratified by education level.

| **SF-36 Norm-based scale** | **Primary school or lower (n=179)** | | | **Secondary education level (n=116)** | | | **Tertiary or higher (n=98)** | | | **p-value**** |
| --- | --- | --- | --- | --- | --- | --- | --- | --- | --- | --- |
|  | **Mean** | **SD** | **Alpha*** | **Mean** | **SD** | **Alpha*** | **Mean** | **SD** | **Alpha*** |  |
| Physical functioning | 55.7 | 3.5 | 0.757 | 56.3 | 2.4 | 0.684 | 56.5 | 1.3 | 0.741 | 0.478 |
| Role physical | 54.6 | 8.8 | 0.699 | 54.5 | 8.5 | 0.645 | 54.8 | 7.2 | 0.7 | 0.555 |
| Role emotional | 52.6 | 11.4 | 0.722 | 53.1 | 10.6 | 0.623 | 53.3 | 8.8 | 0.665 | 0.859 |
| Energy/vitality | 61.5 | 6.3 | 0.726 | 62.5 | 6.1 | 0.632 | 61.4 | 6.2 | 0.715 | 0.353 |
| Emotional well-being | 55.1 | 6.5 | 0.749 | 55.6 | 7 | 0.647 | 54.4 | 7.7 | 0.73 | 0.505 |
| Social functioning | 53.1 | 7.2 | 0.679 | 53.4 | 6.8 | 0.581 | 54.9 | 4.6 | 0.653 | 0.166 |
| Pain | 59 | 7.8 | 0.691 | 59 | 7.2 | 0.56 | 59.4 | 8.3 | 0.702 | 0.231 |
| General health | 48.7 | 7.1 | 0.751 | 49.4 | 7.9 | 0.706 | 50.8 | 8 | 0.748 | 0.007 |
|  |  |  |  |  |  |  |  |  |  |  |
| Mental health component summary (MCS) | 54.6 | 7.2 | n/a | 55.1 | 7.2 | n/a | 54.5 | 6.7 | n/a | 0.074 |
| Physical health component summary (PCS) | 55.3 | 5.7 | n/a | 55.5 | 5.1 | n/a | 56.3 | 4.4 | n/a | 0.462 |

*For the Alpha, Cronbach’ alpha value of >0.70 was used to define good internal consistency of the SF-36 domains [24].

**For the p value, this was to indicate the difference between means for participants stratified by education. P value <0.05 was a cut-off for significance.

**Supplementary Table 2.** Reliability, central tendency, and variability of scales among participants, stratified by employment status.

| **SF-36 Norm-based scale** | **Employed (n=279)** | | | **Unemployed or student (n=101)** | | | **Other (n=9)** | | | **p-value**** |
| --- | --- | --- | --- | --- | --- | --- | --- | --- | --- | --- |
|  | **Mean** | **SD** | **Alpha*** | **Mean** | **SD** | **Alpha*** | **Mean** | **SD** | **Alpha*** |  |
| Physical functioning | 66.0 | 2.7 | 0.730 | 56.1 | 2.9 | 0.723 | 56.6 | 0.9 | 0.438 | 0.718 |
| Role physical | 54.7 | 8.0 | 0.662 | 54.8 | 7.9 | 0.732 | 51.4 | 13.1 | 0.439 | 0.373 |
| Role emotional | 53.0 | 10.5 | 0.679 | 52.8 | 10.5 | 0.698 | 55.9 | 6.5 | 0.449 | 0.581 |
| Energy/vitality | 619 | 6.0 | 0.693 | 61.9 | 6.5 | 0.709 | 58.1 | 9.3 | 0.129 | 0.396 |
| Emotional well-being | 55.1 | 6.9 | 0.701 | 55.4 | 6.6 | 0.742 | 53.9 | 9.1 | 0.094 | 0.904 |
| Social functioning | 53.6 | 6.7 | 0.643 | 54.4 | 5.4 | 0.667 | 48.4 | 9.9 | 0.794 | 0.016 |
| Pain | 59.4 | 7.3 | 0.665 | 59.3 | 7.2 | 0.652 | 53.9 | 12.9 | 0.567 | 0.089 |
| General health | 49.6 | 7.6 | 0.733 | 48.8 | 7.8 | 0.729 | 52.9 | 6.7 | 0.679 | 0.237 |
|  |  |  |  |  |  |  |  |  |  |  |
| Mental health component summary (MCS) | 54.7 | 7.1 | n/a | 55.1 | 6.9 | n/a | 53.4 | 7.4 | n/a | 0.508 |
| Physical health component summary (PCS) | 55.7 | 5.1 | n/a | 55.5 | 5.1 | n/a | 53.4 | 7.9 | n/a | 0.601 |

*For the Alpha, Cronbach’ alpha value of >0.70 was used to define good internal consistency of the SF-36 domains [24].

**For the P value, this was to indicate the difference between means for participants stratified by employment. P value <0.05 was a cut-off for significance.
